# Supplementary material for: HAT: haplotype assembly tool using short and error-prone long reads
Source: Bioinformatics. 2022 Oct 29;38(24):5352–9. doi: 10.1093/bioinformatics/btac702 (PMC9750119; doi:10.1093/bioinformatics/btac702)
Supplement: btac702_Supplementary_Data [file btac702_supplementary_data.docx]

# Supplemental material -- HAT: Haplotype Assembly Tool using short and long reads

Ramin Shirali Hossein Zade^1^, Aysun Urhan^1,2^, Alvaro Assis de Souza^1^, Akash Singh^1^, and Thomas Abeel^1,2,*^

^1^Delft Bioinformatics Lab, Delft University of Technology Van Mourik, Broekmanweg 6, 2628 XE, Delft, The Netherlands, ^2^Infectious Disease and Microbiome Program, Broad Institute of MIT and Harvard, 415 Main Street, Cambridge, MA, 02142, US.

*To whom correspondence should be addressed.

# Supplemental material

| **Supplementary Table 1.**Haplogenerator parameters for simulating datasets. | | | | | | |
| --- | --- | --- | --- | --- | --- | --- |
| Dataset | SNP | | Insertion | | Deletion | |
|  | Mean | STD | Mean | STD | Mean | STD |
| Triploid low  heterozygosity | 3 | 2.2 | 7 | 2.8 | 9 | 3 |
| Triploid high  heterozygosity | 3 | 2 | 7 | 2 | 9 | 3 |
| Tetraploid low  heterozygosity | 3 | 2.4 | 7 | 2 | 9 | 12 |
| Tetraploid high  heterozygosity | 3 | 1.6 | 6 | 2 | 9 | 12 |
| Pentaploid low  heterozygosity | 3 | 2.4 | 7 | 2 | 9 | 3 |
| Pentaploid high  heterozygosity | 3 | 2 | 7 | 2 | 9 | 12 |

| **Supplementary Table 2.** : Parameters of the tools we use in this study. | | |
| --- | --- | --- |
| Tool name | Parameter name | Parameter value |
| ART | Read length | 125 |
|  | Mean insertion size | 400 |
|  | Standard deviation of insertion size | 20 |
|  | coverage | 20 |
| Badread | quantity | 20 |
| Minimap2 | Secondary | No |
| BWA mem | Default | - |
| Vcffilter | -f | TYPE = SNP |
| FreeBayes | Ploidy | Ploidy of the dataset |
|  | Mean alternate count | 5 |
| Miniasm | Default | - |
| Pilon | Default | - |
| Whatshap haplotag | --ploidy | Ploidy of the chromosome |

| **Supplementary Table 3.** The haplotype reconstruction improvement after each step: we ran HAT on Chromosome ScII of CBS1483 and calculated the number of blocks and number of variants after each iteration of the iterative part of HAT. For all ploidy blocks HAT converges in at most 4 iterations. | | | | | | | | | | |
| --- | --- | --- | --- | --- | --- | --- | --- | --- | --- | --- |
| Ploidy block | Initialization | | Iteration 1 | | Iteration 2 | | Iteration 3 | | Iteration 4 | |
|  | # of blocks | # Phased variants | # blocks | # Phased variants | # blocks | # Phased variants | # blocks | # Phased variants | # blocks | # Phased variants |
| 4473,6122 | 3 | 7 | 3 | 7 | 3 | 7 | Converge | | | |
| 153738,163604 | 8 | 16 | 6 | 18 | 1 | 29 | 1 | 29 | Converge | |
| 171517,197986 | 19 | 38 | 14 | 50 | 3 | 83 | 3 | 83 |  |  |
| 213369,231934 | 12 | 24 | 9 | 27 | 1 | 41 | 1 | 41 |  |  |
| 246873,265346 | 16 | 32 | 8 | 37 | 1 | 62 | 1 | 63 | 1 | 63 |
| 271864,274237 | 2 | 4 | 2 | 5 | 1 | 7 | 1 | 7 | Converge | |
| 284309,287742 | 4 | 8 | 3 | 9 | 1 | 12 | 1 | 12 |  |  |
| 294099,298274 | 8 | 16 | 4 | 23 | 1 | 33 | 1 | 33 |  |  |
| 308600,325784 | 26 | 52 | 13 | 71 | 1 | 93 | 1 | 93 |  |  |
| 334284,334379 | 1 | 2 | 1 | 2 | 1 | 2 | Converge | | | |
| 344243,344336 | 1 | 2 | 1 | 2 | 1 | 2 |  |  |  |  |
| 450369,450450 | 1 | 2 | 1 | 2 | 1 | 2 |  |  |  |  |
| 766447,766450 | 1 | 2 | 1 | 2 | 1 | 2 |  |  |  |  |
| 787679,795363 | 9 | 18 | 5 | 22 | 3 | 31 | 3 | 31 | Converge | |
| 800096,804047 | 1 | 2 | 1 | 2 | 1 | 8 | 1 | 8 |  |  |
| 805511,810793 | 3 | 7 | 3 | 8 | 2 | 13 | 2 | 13 |  |  |

| **Supplementary Table 4.**Real and simulated long reads error rate. The error rates are calculated based on the alignment of reads to the dataset reference genome and samtools stats. | |
| --- | --- |
| Dataset | Error rate percentage |
| CBS1483 real dataset | 15.15 % |
| GB54 real dataset | 14.71 % |
| Triploid low heterozygosity | 12.82 % |
| Triploid high heterozygosity | 12.84 % |
| Tetraploid low heterozygosity | 12.61 % |
| Tetraploid high heterozygosity | 13.07 % |
| Pentaploid low heterozygosity | 12.73 % |
| Pentaploid high heterozygosity | 12.94 % |

| 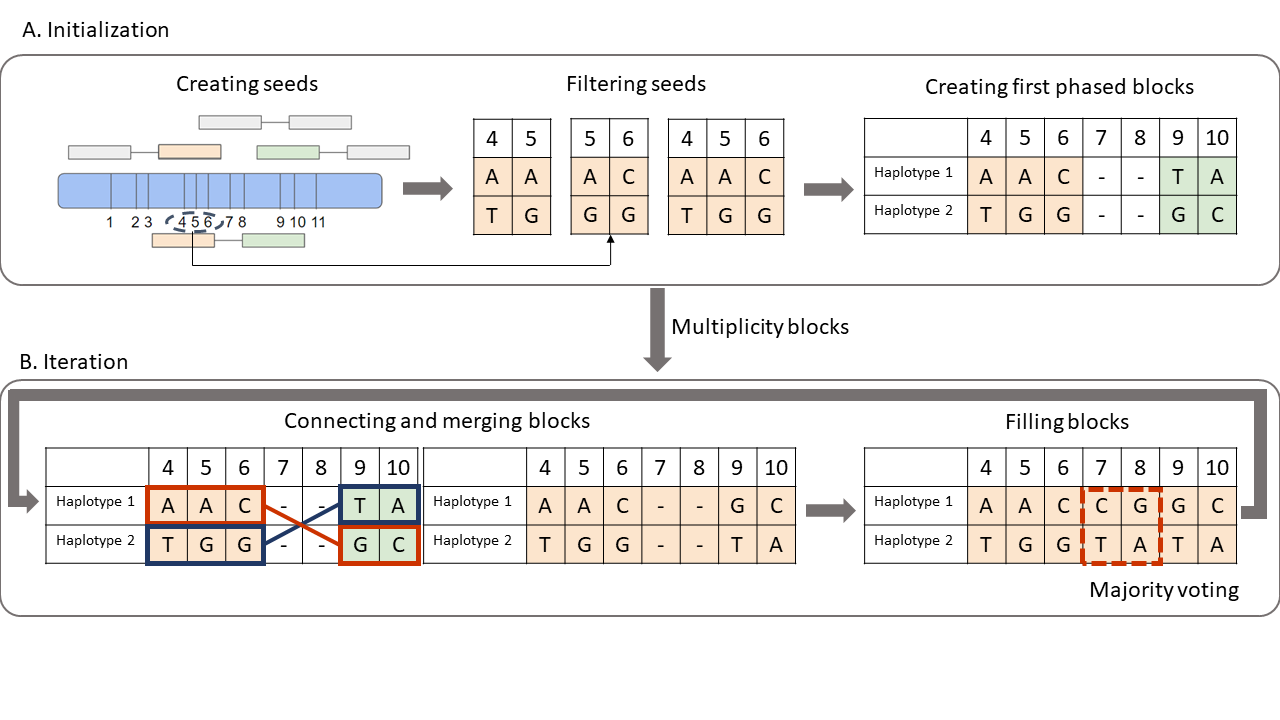 |
| --- |
| **Supplementary Fig. 1**. A. Based on the alignment of the reads that are covering SNPs 4,5 and 6, HAT creates three seeds. In this scenario, because the support of the combinations of alleles of these three seeds are equal, HAT keep the longer one in the filtering seeds step. After that, HAT use the remaining seeds and the combinations of alleles to create first phased blocks. B. Based on the read assignment, the reads that belong to haplotype 1 of block 1, also belong to haplotype 2 of block 2. This means, these two haplotypes are the same, and in the connecting and merging blocks, a bigger block is created, and the mentioned haplotypes are linked together. After that, based on the assignment of reads to each haplotype, and a majority voting between those reads, HAT finds the allele of the unphased SNPs. |

| 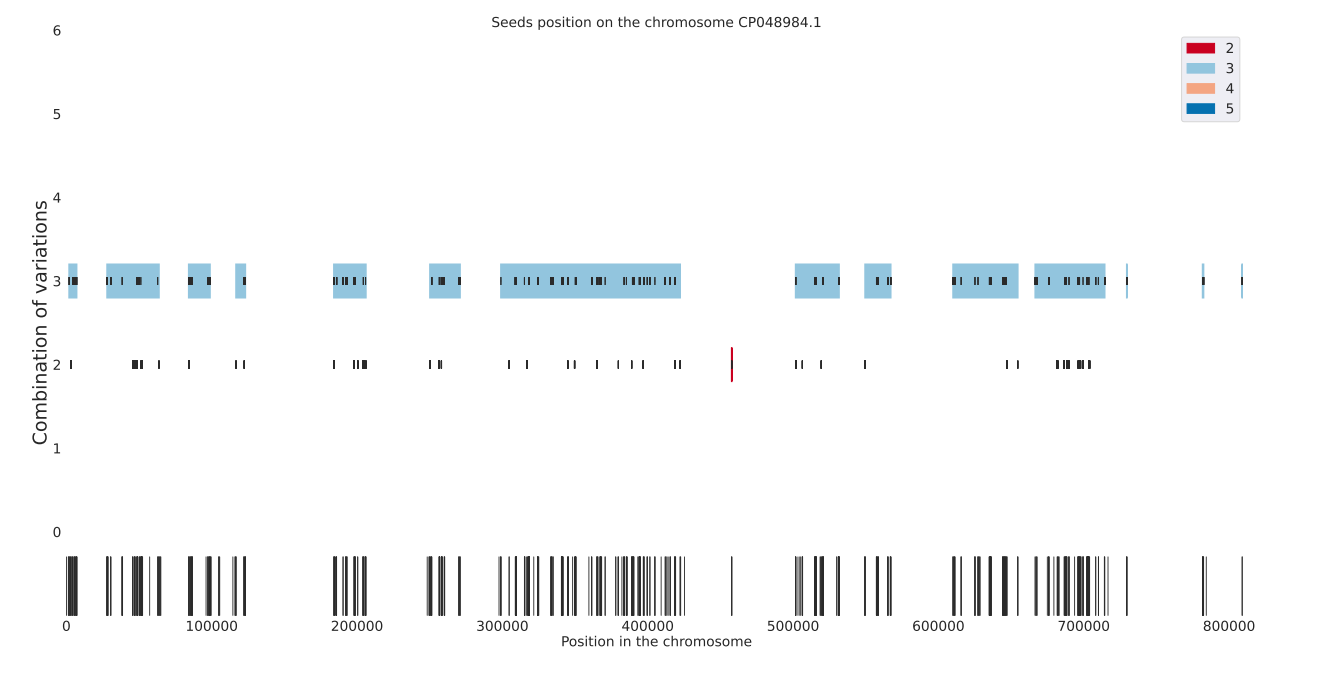 |
| --- |
| **Supplementary Fig. 2**. Multiplicity blocks of Triploid low heterozygosity dataset. |

|  |
| --- |
| **Supplementary Fig. 3**.  Multiplicity blocks of triploid ChrSc2 of CBS1483. |

| 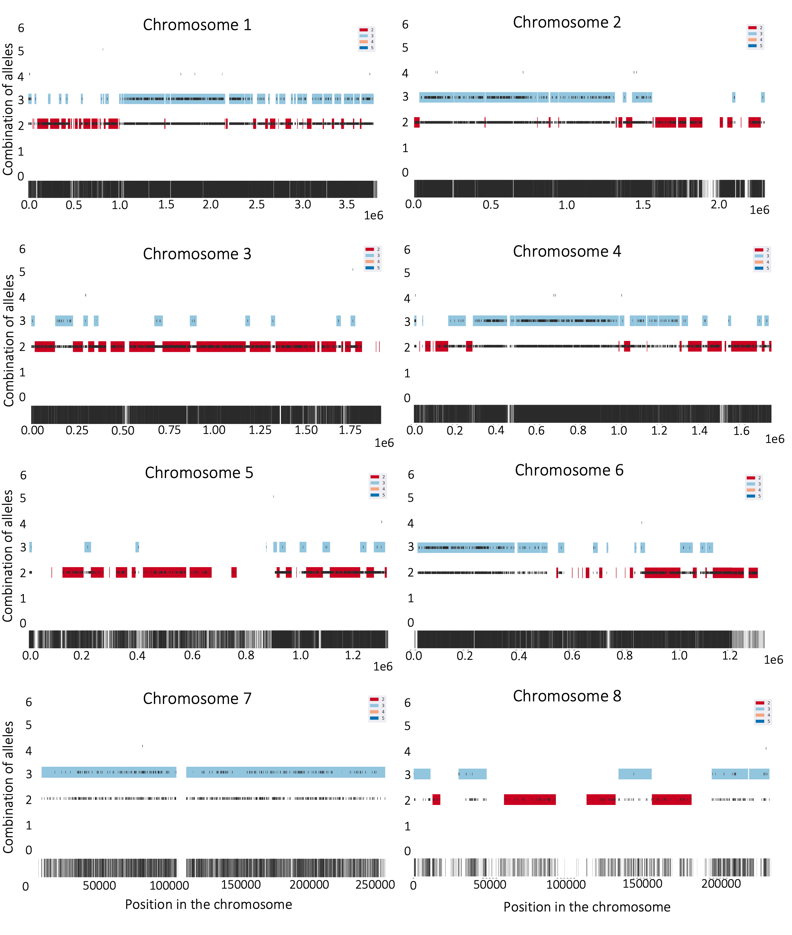 |
| --- |
| **Supplementary Fig. 4**.  Multiplicity blocks of all chromosomes of triploid GB54. |
| 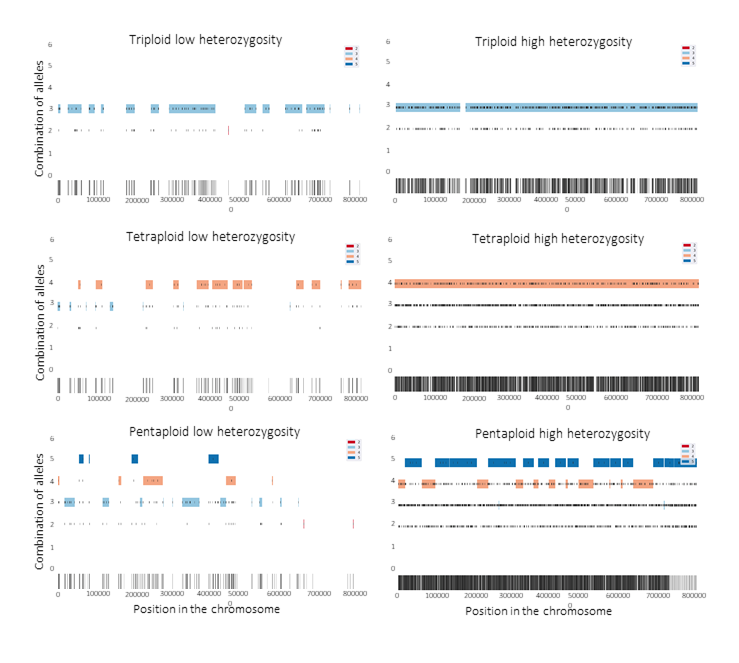 |
| **Supplementary Fig. 5**.  Multiplicity blocks of all simulated datasets. |
